# Urine Assay to Measure Tenofovir Concentrations in Patients Taking Tenofovir Alafenamide

**DOI:** 10.3389/fphar.2020.00286

**Published:** 2020-03-19

**Authors:** Linden Lalley-Chareczko, Emily Hiserodt, Ganesh Moorthy, Athena Zuppa, Karam Mounzer, Helen Koenig

**Affiliations:** ^1^Philadelphia FIGHT Community Health Centers, Philadelphia, PA, United States; ^2^Department of Anesthesiology and Critical Care Medicine, The Children's Hospital of Philadelphia, Philadelphia, PA, United States; ^3^The Perelman School of Medicine at the University of Pennsylvania, Philadelphia, PA, United States

**Keywords:** PrEP, HIV - human immunodeficiency virus, tenofovir alafenamide (TAF), tenofovir, therapaeutic drug monitoring

## Abstract

**Background:** HIV pre-exposure prophylaxis (PrEP) with tenofovir/emtricitabine is effective when taken daily. Previously, we developed a urine assay capable of detecting the prodrug tenofovir (TFV) in patients taking tenofovir disoproxil fumarate (TDF)-based PrEP. However, tenofovir alafenamide (TAF) has replaced TDF due to its different safety profile for HIV treatment and was recently approved as PrEP. Given the need to ensure the aforementioned assay remains available for the purpose of objective adherence monitoring, it is critical to ensure its accuracy for detecting TFV in patients taking TAF.

**Methods:** Blood and urine samples were collected from 3 cohorts of patients: (1) 10 participants living with HIV (PLWH) with suppressed virus on a TAF-based regimen, (2) 10 HIV-participants administered 1 dose of TAF/FTC followed by urine and plasma sampling for 7 days starting 1–3 h post-dose, and (3) 10 HIV-participants administered 7 doses of TAF/FTC followed by urine and plasma sampling for 10 days starting 1–3 h after the last dose. Samples were analyzed using liquid chromatography-tandem mass spectrometry (LC-MS/MS) with high sensitivity and specificity for TFV. HIV-samples were compared to a historical cohort administered one dose of TDF/FTC.

**Results:** PLWH were 90% male, 40% African American, and 10% Hispanic (mean age = 57 y; SD 8.88 y). HIV-participants were 55% male and 70% Caucasian (mean age = 31.6 y; SD 7.70 y). Samples from PLWH demonstrated TFV concentrations 2 logs higher in urine than plasma (1,000 ng/mL vs ±10 ng/mL) at the time of collection. Urine samples following a single dose of TAF in HIV-participants yielded TFV concentrations ranging from 100 to 1,000 ng/mL 1–3 h post-dose and remained >100 ng/mL for 6 days in 8 of 10 participants. Urine samples collected after 7 consecutive doses of TAF yielded TFV concentrations >1,000 ng/mL 1–3 h after dosing discontinuation, with TFV concentrations >1,00 ng/mL 7 days post discontinuation in 8 of 10 participants. Urine TFV concentrations following TAF administration were comparable to those from a historical cohort administered TDF/FTC. Plasma TFV concentrations were low(±10 ng/mL) in both HIV-cohorts at all time points.

**Conclusions:** TFV persists in urine at detectable concentrations in participants taking TAF/FTC for at least 7 days despite largely undetectable plasma concentrations, with urine TFV concentrations comparable to patients taking TDF/FTC. This study demonstrates the ability of a urine TFV assay to measure recent TAF adherence.

## Introduction

Pre-exposure prophylaxis (PrEP) with emtricitabine/tenofovir disoproxil fumarate (FTC/TDF) is highly effective in preventing HIV when taken daily (Garcia-Lerma et al., 2010; Grant et al., 2010; Myers and Mayer, 2011; Prejean et al., 2011; Baeten et al., 2012; Thigpen et al., 2012; Choopanya et al., 2013; Centers for disease control and prevention, 2014; Van Laarhoven et al., 2017), but patient self-report and pill counts are unreliable methods for monitoring adherence (Mimiaga et al., 2009; Poynten et al., 2010). Young men of color who have sex with men (yMSMc) and transgender women (TGW) often struggle to maintain adherence to PrEP (Mimiaga et al., 2009; Poynten et al., 2010; Brinker et al., 2014; Grant et al., 2014) despite high levels of self-reported adherence to PrEP (Hosek et al., 2012), and are heavily impacted by new HIV infections (Brunen et al., 2011; CDC, 2017; Hiemke, 2017).

How to accurately identify suboptimal adherence and develop targeted, strategic interventions to maintain necessary adherence levels for PrEP effectiveness represents a key gap in implementing this otherwise highly effective prevention therapy. Tenofovir (TFV) measurement in urine using liquid chromatography and tandem mass-spectrometry (LC-MS/MS) is a non-invasive and commercially available tool that can be used currently for objective monitoring of people taking FTC/TDF-based PrEP. In patients taking TDF-based regimens, it has been demonstrated that TFV concentrations can be reliably measured in urine, that urine TFV concentrations correlate well with plasma concentrations, and TFV detection in urine reflects medication usage over a window of 1 to at least 7 days after oral FTC/TDF ingestion (Koenig et al., 2017). In a 24-week study of 10 HIV-negative subjects receiving daily FTC/TDF for PrEP, urine TFV concentration >1,000 ng/mL was highly predictive of presence of TFV in plasma (>10 ng/mL) (PPV 0.95, 95%CI, 0.82–0.99; NPV 0.79, 95%CI, 0.49–0.95), suggesting that the urine assay could clearly identify patients who had not taken medication within the previous 48 h (i.e., recent adherence) as their urine TFV concentrations were <1,000 ng/mL, patients who had not taken any medication in the previous 7 days as their urine TFV concentrations were 0 ng/mL, as well as provide some information about intermittent/suboptimal dosing within the previous 7–10 days (>10 to >100 ng/mL; Koenig et al., 2017). Urine TFV testing in this context has also been shown to closely correlate with the research gold standard for objective adherence monitoring, dried blood spot (DBS; Patel et al., 2017), and is preliminarily highly acceptable to yMSMc, particularly among adolescents and young adults (Wertheimer et al., 2006; Liu et al., 2014; Koenig et al., 2017; Hunt et al., 2019). Additionally, urine TFV assessment fills a gap left by plasma, DBS, and hair assessments by providing information about medication adherence over at least a 7 day period: single plasma concentrations only reflect a small window of exposure (2–3 days; Clevenbergh et al., 2002; Nettles et al., 2006; Castillo-Mancilla et al., 2013), and hair analysis and DBS reflect average drug exposures over 1–3 months (Garrett et al., 2019; Hare et al., 2019). In yMSMc, a population known to struggle with adherence (Mimiaga et al., 2009; Poynten et al., 2010), current (previous week) non-adherence data may have greater value than average non-adherence over the prior 3 months given increased vulnerability to HIV exposure, and may create a greater number of opportunities for clinicians to reinforce PrEP adherence behaviors (Koenig et al., 2017; Hunt et al., 2019).

Tenofovir alafenamide (TAF) is replacing TDF as an equally effective tenofovir prodrug in HIV treatment regimens, i.e., TAF/FTC/EVG/COBI, TAF/RPV/FTC, and FTC/TAF and has recently been FDA approved as an alternative oral PrEP agent (Garrett et al., 2019; Hare et al., 2019). When compared to standard dose TDF (300 mg/daily), TAF, at a dose of 25 mg/day, has a 7-fold higher peripheral blood mononuclear cell intracellular tenofovir diphosphate concentration, with only~10% of the plasma tenofovir exposure. At steady state, 25 mg of TAF yielded mean TFV plasma exposures [area under the plasma concentration-time curve (AUCtau)] of 86% lower as compared with the TFV exposures observed with 300 mg of TDF. Increased intracellular concentrations may translate into FTC/TAF's greater antiviral efficacy, a higher barrier to resistance, and an improved safety profile relative to TDF (Ray et al., 2016). Recent findings from the DISCOVER trial, in which 5,387 at-risk adults were randomized to daily FTC/TAF vs. daily FTC/TDF, indicate non-inferiority of FTC/TAF as PrEP relative to FTC/TDF, with a significantly lower overall seroconversion rate than anticipated by investigators (0.26/100PY; Hare et al., 2019).

Given these data, the primary objective of the present study was to determine how long TFV is excreted in the urine of participants who have taken one dose or seven daily doses of FTC/TAF. Based on the pharmacokinetics of TAF, we hypothesized cut-offs indicative of no/intermittent/recent adherence in patients on TAF-based regimens would be approximately 1 log (or 10-fold) lower than those in patients taking TDF-based regimens. Accordingly, we hypothesized the TFV concentration in urine associated with concentrations in plasma for patients on TAF-based regimens would be 100 ng/mL instead of 1,000 ng/mL (i.e., 1 log lower). We also hypothesized that TFV detectability would persist at least 1–2 days longer in the urine of participants dosing at steady state vs. those with limited FTC/TAF exposure.

## Methodology

### Study Setting

Participant recruitment/enrollment and sample collection were conducted at Philadelphia FIGHT Community Health Centers, an urban community-based federally qualified health center. Urine and plasma TFV analyses were performed by the Children's Hospital of Philadelphia (CHOP) Pharmacology Research Unit. Urine samples were sent to a local, commercial laboratory for assessment of specific gravity, urine creatinine, and pH to control for inter-subject variability.

### Study Design and Participant Recruitment

This study employed a sequential, 3-cohort design with a sample size of 10 for each cohort (total *n* = 30). All participants were 18 years of age or older and able to provide written informed consent in English. PLWH were recruited via electronic medical record prescreening and face-to-face requests during clinic visits to determine interest in the study. HIV-negative participants were recruited by flyers and word of mouth from FIGHT associated clinics and the surrounding community. This study was approved by the Institutional Review Board at Philadelphia FIGHT.

The first cohort employed both a qualitative and semi-quantitative evaluation of the relationship between urine and plasma TFV in 10 PLWH with undetectable viral loads for greater than 12 weeks prior to consent per available medical records and a recent undetectable viral load in the previous 4 weeks on an antiretroviral regimen containing FTC/TAF (i.e., Genvoya™, Odefsey™, or Descovy™ in combination with another HIV medication or medications). Participants returned to the clinic at their convenience and underwent a one-time, pre-dose urine and plasma collection for TFV concentration analyses, reflecting drug concentrations ~24 h after last medication ingestion. Participants also kept a daily diary of FTC/TAF dosing for the 3 days prior to sample collection.

The second and third cohorts enrolled HIV negative participants who were given either a single dose (cohort 2) or 7 daily doses (cohort 3) of FTC/TAF, with the study design of the 7 dose cohort based on data showing that 7 daily doses of FTC/TDF achieves therapeutic drug concentrations consistent with protection from HIV (Wertheimer et al., 2006). These cohorts underwent additional laboratory screenings for acute or chronic hepatitis B infection, renal dysfunction (Creatinine Clearance <50 mL/min by Cockroft-Gault equation), and/or DAIDS grade 3 laboratory abnormality at screening, and were asked to report any history of severe infections requiring treatment such as tuberculosis, bone fractures not explained by trauma and/or a known allergy/sensitivity to the study FTC/TAF or its components in accordance with standard of care practices when prescribing FTC/TAF. Concurrent participation in an HIV vaccine study or concurrent use of any other antiretroviral agent were also assessed by participant self-report. Morning urine and plasma samples were then collected starting 1 h post-dose and for 6 (cohort 2) or 9 (cohort 3) consecutive days thereafter.

### Sample Processing

Urine and plasma samples were stored at −78°C until analysis. TFV concentrations are stable at least for 48 weeks at −78°C (Lalley-Chareczko et al., 2018). Urine samples were diluted 50-fold in blank plasma, extracted and then analyzed by LC-MS/MS. Pre-dilution of urine samples with blank human plasma was utilized as an approach to minimize the impact of urine characteristics in causing variability in TFV concentration measurements. Plasma samples were analyzed without dilution. The range of concentration in diluted urine samples were below lower limit of quantitation (10 ng/mL) to 500 ng/mL (assay range: 10–10,000 ng/mL; Koenig et al., 2017).

## Results

**Figure 1:**
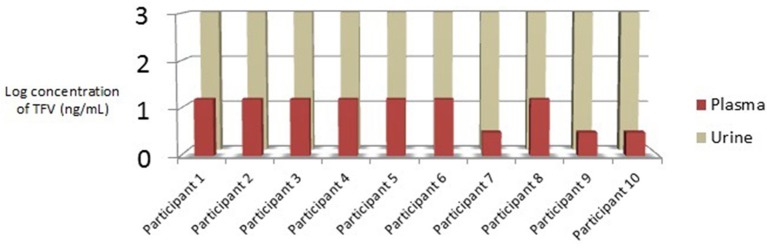
Cohort 1: Relationship between urine and plasma TFV concentrations in PLWH taking daily TAF. TFV, tenofovir; PLWH, Participants Living with HIV; TAF, tenofovir alafenamide; ng/mL, nanograms per millimeter.

Cohort 1 (Participants living with HIV): PLWH were 90% male, 10% female, 40% African American, 60% Caucasian, and 10% Hispanic. The median age was 53.5 years, with a range of 51 to 79 years (Table 1). Recorded HIV treatment regimens in this cohort included FTC/TAF plus one of the following: dolutegravir (3), boosted elvitegravir (3), boosted darunavir (2), raltegravir (1), or rilpivirine (1). Urinalysis laboratory results were collected for future studies and are listed in Appendix A in Supplementary Material. Urine and plasma samples from PLWH were collected between 13.5 and 28.3 h after the last TAF containing medication dose as per participant report (m = 20.2 h); urine and plasma samples were collected, on average, 4.4 min apart (min = 1 min; max = 14 min). Urine samples from PLWH demonstrated TFV concentrations 2 logs higher than plasma (>1,000 ng/mL vs. ±10 ng/mL, respectively); plasma TFV concentrations were low (70% >10 ng/mL; 30% <10 ng/mL) for all PLWH (Figure 1).

**Table 1:** Participant demographics.

	**Cohort 1**	**Cohort 2**	**Cohort 3**
Mean age (range)	57 (51–79) years	33 (23–47) years	30 (23–43) years
**Race % (*****n*****)**			
Caucasian	60% (6)	70% (7)	60% (6)
Black/African American	40% (4)	20% (2)	20% (2)
Asian	0	10% (1)	10% (1)
Other	0	0	10% (1)
**Ethnicity % (*****n*****)**			
Hispanic	10% (1)	0	0
Non-hispanic	90% (9)	100% (10)	100% (10)
**Gender % (*****n*****) No participants identified as transgender**			
Male	90% (9)	60% (6)	50% (5)
Female	10% (1)	30 (3)	40% (4)
Gender Non-conforming	0	10% (1)	10% (1)

Cohorts 2 & 3: HIV-negative participants, collectively, were 55% male, 45% female, 65% Caucasian, 20% Black/African American, 10% Asian, and 5% endorsing other racial backgrounds. The median age for HIV-negative patients was 30.5 years with a range of 23–47 years (Table 1). As in Cohort 1, urinalysis results are listed in Appendix A in Supplementary Material.

**Figure 2:**
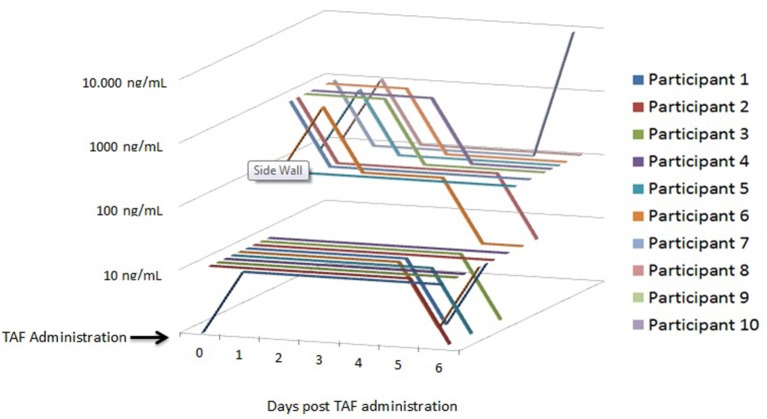
Cohor t 2: Urine/Plasma TFV Concentrations following a Single Dose of FTC/TAF in 10 HIV-Participants. TFV, tenofovir; FTC/TAF, emtricitabine/tenofovir alafenamide; TAF, tenofovir alafenamide; ng/mL, nanograms per millimeter.

Cohort 2: Urine samples collected following a single dose of FTC/TAF in all 10 HIV-negative participants yielded TFV semi-quantitative concentrations ranging from 100 to >1,000 ng/mL 1–3 h post-dose, with 6 of 10 participant samples (60%) demonstrating TFV concentrations >1,000 ng/mL at that time. At 24 h post-dose, 6 of 10 participant samples had urine TFV concentrations >1,000 ng/mL; however, samples did not necessarily come from the same participants as those who had TFV concentrations >1,000 ng/mL 1–3 h post-dose. For example, participant #1 had TFV concentrations >100 ng/mL 1–3 h post-dose and 24 h post-dose, whereas participants 2, 7, and 10 had TFV concentrations increase from >100 ng/mL 1–3 h post-dose to >1,000 ng/mL 24 h post-dose. Participants 3, 4, and 9 had urine TFV concentrations >1,000 ng/mL 1–3 h post-dose that fell to >100 ng/mL 24 h post-dose. Participants 5, 6, and 8 had urine TFV concentrations that remained consistent at >1,000 ng/mL from 1–3 to 24 h post-dose. Urine TFV concentrations observed over the remaining 5 days of collection displayed a downward trend with the same variability described above (Table 2; Figure 2).

**Table 2:** Observed urine concentrations from the single dose cohort.

**Days post TAF dosing**	**% Urine TFV >10,000 ng/mL**	**% Urine TFV >1,000 ng/mL**	**% Urine TFV >100 ng/mL**	**% Urine TFV >10 ng/mL**	**% Urine TFV <10 ng/mL**
0	–	60	40	–	–
1	–	60	40	–	–
2	–	30	70	–	–
3	–	20	80	–	–
4	–	–	100	–	–
5	–	–	90	10	–
6	10^*^	–	70	10	10

*TFV, tenofovir; HIV+, Human Immunodeficiency Virus-Positive; TAF, tenofovir alafenamide; ng/mL, nanograms per milliliter*.

* *Verified upon repeat analysis*.

**Figure 3:**
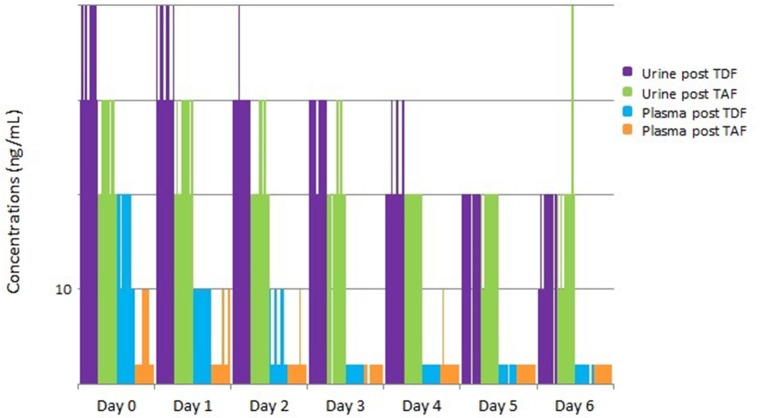
Urine/Plasma TFV concentrations after a single dose of FTC/TAF in 10 HIV-subjects, with comparison to historical cohort of subjects given a single dose of FTC/TDF. TFV, tenofovir; FTC/TAF, emtricitabine/tenofovir alafenamide; HIV–, Human Immunodeficiency Virus-Negative; FTC/TDF: emtricitabine/tenofovir disoproxil fumarate; TDF, tenofovir disoproxil fumarate; TAF, tenofovir alafenamide; ng/mL, nanograms per millimeter.

These concentrations were lower than those from a historical cohort administered FTC/TDF (Koenig et al., 2017); urine TFV concentrations rose more rapidly after medication ingestion in subjects receiving FTC/TDF and were, on average, higher for the first 4 days after discontinuation of medication compared to those receiving FTC/TAF (Figure 3). Sixty percent of samples from participants dosed with FTC/TDF displayed urine TFV concentrations >10,000 ng/mL 1–3 and 24 h after dosing, whereas 60% of participants reached the >1,000 ng/mL concentration 1–3 and 24 h after a single FTC/TAF dose. One hundred percent of patients dosed with FTC/TDF displayed urine TFV concentrations >1,000 ng/mL through the second day post dose, and 90% continued to display this concentration on the 3rd sampling day. However, after a single FTC/TAF dose, TFV concentrations dropped below 1,000 ng/mL in 70% of samples 2 days after dosing and 80% of samples 3 days after dosing (Figure 3).

Plasma concentrations from the single-dose cohort remained low throughout sample collection, as expected given the plasma penetration of TAF. Plasma TFV concentrations 1–3 h post-dose were undetectable in 1 subject (10%), detectable but <10 ng/mL in 5 (50%) subjects, and <100 ng/mL in 4 (40%) of subjects, with plasma TFV concentrations falling quickly over subsequent collections (Figure 3). These concentrations are low in comparison to the historical TDF cohort where 70% of samples displayed >100 ng/mL 1–3 h after dosing and 100% maintained low-level detectability 24 h after dosing.

**Figure 4:**
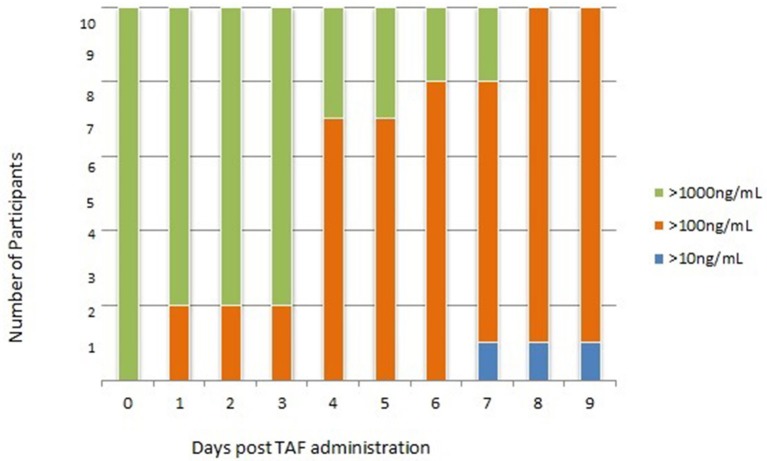
Cohort 3: Urine TFV Concentrations following 7 consecutive doses of FTC/TAF in 10 HIV-subjects. TFV, tenofovir; FTC/TAF, emtricitabine/tenofovir alafenamide; HIV–, Human Immunodeficiency Virus-Negative; TAF, tenofovir alafenamide; ng/mL, nanograms per millimeter.

Cohort 3: Urine samples collected after 7 consecutive doses of FTC/TAF (steady state dosing) yielded TFV concentrations >1,000 ng/mL 1–3 h after discontinuation of dosing in all 10 participants with TFV concentrations remaining >1,000 ng/mL in 80% samples collected 24 after dosing discontinuation. As in the single dose cohort, urine TFV concentrations remained detectable, with 80% samples yielding TFV concentrations >1,000 ng/mL at 2 and 3 days post dosing discontinuation; however, these were not necessarily the samples coming from the same participant, as described above. As in the single-dose cohort, participant samples displayed a downward trend with variability similar to the single-dose cohort over the remaining 6 days of sample collection (Table 3; Figure 4). In comparison to subjects who took a single dose of FTC/TAF, participants at steady state demonstrated urine TFV concentrations that started higher immediately post-dose, and remained higher at all measured time points, reflecting a longer “look-back period” (period of time from when sample is collected that a clinician may have insight into recent dosing, i.e., in this case 7–10 days) in participants taking daily FTC/TAF. Plasma samples collected after 7 consecutive doses of FTC/TAF yielded TFV concentrations similar to those observed in the single-dose cohort.

**Table 3:** Observed urine concentrations from the seven dose cohort.

**Days post TAF dosing**	**% Urine TFV >10,000 ng/mL**	**% Urine TFV >1,000 ng/mL**	**% Urine TFV >100 ng/mL**	**% Urine TFV >10 ng/mL**	**% Urine TFV <10 ng/mL**
0	–	100	–	–	–
1	–	80	20	–	–
2	–	80	20	–	–
3	–	80	20	–	–
4	–	30	70	–	–
5	–	30	70	–	–
6	–	20	80	–	–
7	–	20	70	10	–
8	–	–	90	10	–
9	–	–	90	10	–

*TFV, tenofovir; HIV+, Human Immunodeficiency Virus-Positive; TAF, tenofovir alafenamide; ng/mL, nanograms per milliliter*.

## Discussion

The primary objective of this study was to determine the pattern of TFV excretion in the urine and plasma of participants taking FTC/TAF. Based on the published pharmacokinetics of TAF, we hypothesized TFV concentrations in the urine and plasma of participants taking FTC/TAF, whether living with HIV or not, would be approximately 1 log (or 10-fold) lower than those observed in a historical cohort of participants taking FTC/TDF, and the TFV concentration in urine associated with concentrations in plasma for participants on TAF-based regimens would be 100 ng/mL instead of 1,000 ng/mL (i.e., 1 log lower).

The results from this study demonstrate that TFV concentrations in urine and plasma samples were indeed approximately 10-fold (1 log) lower in patients taking FTC/TAF relative to those taking FTC/TDF, with only 60% of participants in the (comparable) single-dose study exceeding the previously established clinical cut-off for recent adherence of 1,000 ng/mL within 24 h after the last FTC/TAF dose. However, in the steady state cohort (cohort 3), urine TFV concentrations were >1,000 ng/mL within 24 h after the last FTC/TAF dose in the majority (80%) of patients. The observed 1 log differential (both in urine and plasma) between TFV from TDF and TAF dosing is expected given the known pharmacokinetics of both drugs. Additionally, both formulations of tenofovir (TDF and TAF) are renally cleared, thus generating higher urine TFV concentrations than plasma.

The present study also compared the excretion pattern of TFV in patients taking daily FTC/TAF for 7 days (i.e., steady state dosing) to the TFV excretion patterns in those who have taken FTC/TAF for extremely short periods of time (i.e., a single dose). TFV remained detectable in the urine of participants exposed to 7 consecutive doses of FTC/TAF for 9 days after the discontinuation of daily dosing, extending the period of detectability from that observed after a single FTC/TAF dose (6 days). Urine TFV concentrations >1,000 ng/mL, suggestive of recent adherence, persisted for 48–96 h for the majority (80%) of participants, similar to TFV concentrations observed in a historical cohorts of patients taking FTC/TDF (Koenig et al., 2017); in comparison, only 30% of urine samples contained concentrations >1,000 ng/mL 48 h after a single dose of FTC/TAF. None of the 7-dose cohort samples reached “undetectability” (<10 ng/mL) in urine during the 10-day observation period. The longer period in which TFV was detected post-dosing in the 7-day cohort relative to the single-dose cohort is likely due to higher drug concentrations achieved through steady state dosing and thus a longer time to clear completely from the urine.

Exploring TFV excretion patterns using a cohort of patients dosing at steady state may provide a more robust understanding of urinary TFV cut-offs indicative of recent PrEP dosing once more information is available about duration of protection after stopping FTC/TAF. For example, MSM taking daily FTC/TDF are considered to have protective TFV concentrations for rectal exposure to HIV until 7 days after last dose (Anderson et al., 2016). If the same is shown to be true for patients taking FTC/TAF, and with FDA approval of FTC/TAF for the purpose of PrEP, then the data from this study may provide a basis for the formulation of standard urinary TFV concentrations consistent with protection against HIV in this population. Furthermore, establishing urine TFV norms for persons taking FTC/TAF for PrEP is an important step for prescribers and providers who order urine TFV testing as a measure of adherence and tailor supportive counseling based on those results.

The present study is limited by a fair degree of subject-to-subject variability in concentrations over the washout period. As a result, it was more difficult to identify indications at time points that would be clinically useful for physicians, compared to those previously established for patients taking FTC/TDF. A next step would include determination of the best way to correct urine TFV values for inter-subject variability by assessing which measure (specific gravity, urine creatinine, pH) will maximize the correlation between urine TFV concentrations and an ideal line of elimination. Secondly, as all 10 participants in the “steady state” cohort still had detectable tenofovir in their urine at the end of the study period (10 days after final dose of FTC/TAF) using uncorrected urine TFV concentrations, we are not able to establish definitive thresholds for imperfect recent adherence (more than 24–48 days post-dose) and non-adherence.

However, at the time of this writing, a fully quantitative assay is being developed and validated by UrSure, Inc, which will be able to provide more specific thresholds for these categories of adherence and may also reveal more nuanced variation between cut-off values for the urine TFV assay in patients taking FTC/TAF vs. those taking FTC/TDF.

This study demonstrates the feasibility of using a urine TFV assay to assess recent adherence to TAF using similar cutoffs to those in patients taking TDF-based regimens and provides proof of concept to further develop this assay for use in patients taking TAF-based regimens. Future efforts will focus on refining these cut-offs for patients taking TDF- and TAF-based regimens using a fully quantitative assay, as well as better addressing the differences in urinary TFV clearance patterns between TDF and TAF-based regimens.

## Data Availability Statement

The datasets generated for this study are available on request to the corresponding author.

## Ethics Statement

The studies involving human participants were reviewed and approved by Philadelphia FIGHT Institutional Review Board. The patients/participants provided their written informed consent to participate in this study.

## Author Contributions

HK and KM were responsible for protocol conceptualization and oversight. LL-C and EH were responsible for participant recruitment, sample collection, and data management. GM and AZ ran sample analyses via liquid chromatography-tandem mass spectrometry. LL-C and HK were primarily responsible for drafting the manuscript, however, all authors participated in revisions and edits.

### Conflict of Interest

Philadelphia FIGHT received research funds from Gilead Sciences for the conduct of this research. Funds provided covered the cost of laboratory analyses, patient stipends, and travel/publication costs. As a funder, Gilead Sciences approved our independently drafted study design, but had no further role in study implementation, data collection and analysis, decision to publish, or preparation of the manuscript. HK, and KM are both paid consultants for Gilead Sciences. HK is also co-founder of UrSure, Inc., developers and manufacturers of urine assays to monitor medication adherence (http://www.ursureinc.com) based in Boston, Massachusetts, USA. The remaining authors declare that the research was conducted in the absence of any commercial or financial relationships that could be construed as a potential conflict of interest.
