# Supplementary material for: Urine Assay to Measure Tenofovir Concentrations in Patients Taking Tenofovir Alafenamide
Source: Front Pharmacol. 2020 Mar 19;11:286. doi: 10.3389/fphar.2020.00286 (PMC7096572; doi:10.3389/fphar.2020.00286)
Supplement: Supplementary file 1 [file Data_Sheet_1.docx]

Appendix A

Urinalysis Results by Cohort

|  | **Cohort 1 (Participants living with HIV on TAF containing treatment regimen)** |  |
| --- | --- | --- |
|  | **Urine Analyte** |  |
| **Participant 1** | Specific Gravity | 1.021 |
|  | Creatinine [mg/dL] | 77.9 |
|  | Microalbumin/Creatinine Ratio [mg/g; creat] | 5.0 |
|  |  |  |
| **Participant 2** | Specific Gravity | 1.018 |
|  | Creatinine [mg/dL] | 104.8 |
|  | Microalbumin/Creatinine Ratio [mg/g; creat] | 5.0 |
|  |  |  |
| **Participant 3** | Specific Gravity | 1.024 |
|  | Creatinine [mg/dL] | 153.7 |
|  | Microalbumin/Creatinine Ratio [mg/g; creat] | 2.7 |
|  |  |  |
| **Participant 4** | Specific Gravity | 1.022 |
|  | Creatinine [mg/dL] | 103.3 |
|  | Microalbumin/Creatinine Ratio [mg/g; creat] | 32.3 |
|  |  |  |
| **Participant 5** | Specific Gravity | 1.022 |
|  | Creatinine [mg/dL] | 159.9 |
|  | Microalbumin/Creatinine Ratio [mg/g; creat] | <1.9 |
|  |  |  |
| **Participant 6** | Specific Gravity | 1.028 |
|  | Creatinine [mg/dL] | 203.1 |
|  | Microalbumin/Creatinine Ratio [mg/g; creat] | 3.4 |
|  |  |  |
| **Participant 7** | Specific Gravity | 1.024 |
|  | Creatinine [mg/dL] | 256.5 |
|  | Microalbumin/Creatinine Ratio [mg/g; creat] | 9.4 |
|  |  |  |
| **Participant 8** | Specific Gravity | 1.015 |
|  | Creatinine [mg/dL] | 119.0 |
|  | Microalbumin/Creatinine Ratio [mg/g; creat] | 61.1 |
|  |  |  |
| **Participant 9** | Specific Gravity | 1.026 |
|  | Creatinine [mg/dL] | 164.1 |
|  | Microalbumin/Creatinine Ratio [mg/g; creat] | <1.8 |
|  |  |  |
| **Participant 10** | Specific Gravity | 1.024 |
|  | Creatinine [mg/dL] | 173.7 |
|  | Microalbumin/Creatinine Ratio [mg/g; creat] | 2.3 |

|  | **Cohort 2 (HIV- participants who took 1 dose of TAF)** |  |  |  |  |  |  |  |
| --- | --- | --- | --- | --- | --- | --- | --- | --- |
|  | **Urine Analyte** | **Days post dose** | | | | | | |
|  |  | **0** | **1** | **2** | **3** | **4** | **5** | **6** |
| **Participant 1** | Specific Gravity | 1.017 | 1.005 | 1.104 | 1.029 | 1.026 | 1.016 | 1.021 |
|  | Creatinine [mg/dL] | 66.6 | 18.5 | 107.9 | 216.9 | 228.3 | 101.5 | 150.4 |
|  | Microalbumin/Creatinine Ratio [mg/g; creat] | <4.5 | 23.8 | 3.6 | 2.5 | 3.6 | 13.1 | 3.7 |
|  |  |  |  |  |  |  |  |  |
| **Participant 2** | Specific Gravity | 1.026 | 1.025 | 1.012 | 1.024 | >1.030 | 1.007 | 1.008 |
|  | Creatinine [mg/dL] | 153.0 | 221.6 | 56.1 | 213.5 | 450.5 | 25.0 | 22.8 |
|  | Microalbumin/Creatinine Ratio [mg/g; creat] | 4.8 | 13.0 | 11.4 | 7.3 | 18.1 | 16.0 | <13.2 |
|  |  |  |  |  |  |  |  |  |
| **Participant 3** | Specific Gravity | 1.012 | 1.007 | 1.020 | TNP | 1.010 | 1.020 | 1.013 |
|  | Creatinine [mg/dL] | 85.2 | 23.3 | 184.0 | TNP | 81.3 | 173.8 | 142.4 |
|  | Microalbumin/Creatinine Ratio [mg/g; creat] | 3.9 | <12.9 | 8.6 | TNP | <3.7 | 24.1 | 7.9 |
|  |  |  |  |  |  |  |  |  |
| **Participant 4** | Specific Gravity | 1.028 | 1.018 | 1.011 | 1.013 | 1.240 | 1.020 | 1.011 |
|  | Creatinine [mg/dL] | 197.2 | 122.4 | 67.1 | 69.1 | 168.4 | 125.5 | 76.2 |
|  | Microalbumin/Creatinine Ratio [mg/g; creat] | 2.4 | <2.5 | <4.5 | <4.3 | 1.9 | <2.4 | 5.8 |
|  |  |  |  |  |  |  |  |  |
| **Participant 5** | Specific Gravity | 1.027 | 1.026 | >1.030 | 1.026 | 1.027 | 1.017 | 1.019 |
|  | Creatinine [mg/dL] | 299.8 | 280.4 | 329.4 | 141.2 | 278.7 | 129.5 | 166.4 |
|  | Microalbumin/Creatinine Ratio [mg/g; creat] | 10.9 | 269.9 | 10.9 | 6.4 | 21.1 | 13.1 | 47.2 |
|  |  |  |  |  |  |  |  |  |
| **Participant 6** | Specific Gravity | 1.022 | 1.026 | 1.024 | 1.024 | 1.022 | 1.015 | 1.022 |
|  | Creatinine [mg/dL] | 193.3 | 277.8 | 355.1 | 339.9 | 190.8 | 79.3 | 229.6 |
|  | Microalbumin/Creatinine Ratio [mg/g; creat] | 45.8 | 294.5 | 156.3 | 77.6 | 15.6 | 6.6 | 23.7 |
|  |  |  |  |  |  |  |  |  |
| **Participant 7** | Specific Gravity | 1.027 | 1.026 | >1.030 | >1.030 | >1.030 | 1.019 | >1.030 |
|  | Creatinine [mg/dL] | 168.0 | 152.7 | 163.2 | 165.7 | 317.6 | 187.7 | 224.1 |
|  | Microalbumin/Creatinine Ratio [mg/g; creat] | 1.9 | 3.1 | 2.1 | 2.1 | 3.1 | 1.9 | 2.0 |
|  |  |  |  |  |  |  |  |  |
| **Participant 8** | Specific Gravity | 1.022 | 1.026 | 1.024 | 1.029 | 1.021 | 1.019 | 1.016 |
|  | Creatinine [mg/dL] | 149.1 | 185.0 | 144.3 | 211.2 | 130.0 | 104.2 | 141.2 |
|  | Microalbumin/Creatinine Ratio [mg/g; creat] | 6.2 | 9.0 | 6.4 | 6.5 | 30.8 | 11.0 | 7.8 |
|  |  |  |  |  |  |  |  |  |
| **Participant 9** | Specific Gravity | 1.015 | 1.009 | 1.008 | 1.027 | 1.024 | 1.027 | 1.012 |
|  | Creatinine [mg/dL] | 68.5 | 61.5 | 43.1 | 193.4 | 104.8 | 195.0 | 60.3 |
|  | Microalbumin/Creatinine Ratio [mg/g; creat] | <4.4 | <4.9 | <7.0 | 2.5 | <2.9 | 17.0 | 6.5 |
|  |  |  |  |  |  |  |  |  |
| **Participant 10** | Specific Gravity | 1.020 | 1.026 | 1.022 | 1.021 | 1.020 | 1.026 | 1.024 |
|  | Creatinine [mg/dL] | 86.3 | 202.5 | 133.8 | 151.0 | 218.3 | 211.3 | 145.5 |
|  | Microalbumin/Creatinine Ratio [mg/g; creat] | <3.5 | 8.0 | 5.0 | 2.4 | 4.4 | 3.6 | 3.8 |

|  | **Cohort 3 (HIV- participants who took 7 doses of TAF)** |  |  |  |  |  |  |  |  |  |  |
| --- | --- | --- | --- | --- | --- | --- | --- | --- | --- | --- | --- |
|  | **Urine Analyte** | **Days post dose** | | | | | | | | | |
|  |  | **0** | **1** | **2** | **3** | **4** | **5** | **6** | **7** | **8** | **9** |
| **Participant 1** | Specific Gravity | 1.026 | 1.030 | 1.030 | 1.030 | 1.023 | 1.028 | 1.030 | 1.030 | 1.022 | 1.030 |
|  | Creatinine [mg/dL] | 162.6 | 225.5 | 177.8 | 210.6 | 103.5 | 205.4 | 213.3 | 235.9 | 184.8 | 183.3 |
|  | Microalbumin/Creatinine Ratio [mg/g; creat] | 1.8 | 1.9 | 2.2 | 2.3 | 3.2 | 3 | 3.3 | 1.6 | 1.6 | 2.5 |
|  |  |  |  |  |  |  |  |  |  |  |  |
| **Participant 2** | Specific Gravity | 1.019 | 1.019 | 1.015 | 1.019 | 1.017 | 1.023 | 1.030 | 1.018 | 1.019 | 1.025 |
|  | Creatinine [mg/dL] | 77.9 | 92.0 | 105.0 | 149.6 | 132.2 | 188.5 | 290.9 | 90.6 | 92.0 | 165.9 |
|  | Microalbumin/Creatinine Ratio [mg/g; creat] | 4.1 | 4.1 | 7.0 | 6.7 | 7.3 | 5.4 | 9.6 | 5.0 | 4.5 | 11.8 |
|  |  |  |  |  |  |  |  |  |  |  |  |
| **Participant 3** | Specific Gravity | 1.008 | 1.012 | 1.025 | 1.024 | 1.015 | 1.014 | 1.013 | 1.026 | 1.013 | 1.027 |
|  | Creatinine [mg/dL] | 59.1 | 160.2 | 213.3 | 141.0 | 91.5 | 74.1 | 92.7 | 158.9 | 90.0 | 144.3 |
|  | Microalbumin/Creatinine Ratio [mg/g; creat] | 8.0 | 7.1 | 3.8 | 3.0 | 6.9 | 5.0 | 3.6 | 4.2 | 3.3 | 2.8 |
|  |  |  |  |  |  |  |  |  |  |  |  |
| **Participant 4** | Specific Gravity | 1.010 | 1.026 | 1.021 | 1.018 | 1.029 | 1.014 | 1.028 | 1.022 | 1.023 | 1.025 |
|  | Creatinine [mg/dL] | 43.0 | 259.7 | 137.6 | 108.0 | 278.5 | 68.2 | 243.6 | 119.7 | 189.9 | 189.3 |
|  | Microalbumin/Creatinine Ratio [mg/g; creat] | <7.0 | 2.2 | 2.2 | 2.8 | 2.5 | 4.4 | 2.1 | 2.5 | 2.7 | 1.9 |
|  |  |  |  |  |  |  |  |  |  |  |  |
| **Participant 5** | Specific Gravity | 1.019 | 1.015 | TNP | 1.019 | 1.018 | 1.019 | 1.017 | 1.019 | 1.020 | 1.017 |
|  | Creatinine [mg/dL] | 128.4 | 126.8 | TNP | 148.1 | 120.8 | 115.4 | 124.9 | 174.4 | 134.1 | 108.2 |
|  | Microalbumin/Creatinine Ratio [mg/g; creat] | 3.7 | 2.7 | TNP | 2.6 | <2.5 | <2.6 | 2.5 | 2.7 | 2.5 | <2.8 |
|  |  |  |  |  |  |  |  |  |  |  |  |
| **Participant 6** | Specific Gravity | 1.014 | 1.017 | 1.009 | 1.015 | 1.007 | 1.017 | 1.014 | 1.01 | 1.007 | 1.013 |
|  | Creatinine [mg/dL] | 80.5 | 199.8 | 27.9 | 71.7 | 22.0 | 85.8 | 28.8 | 33.5 | 6.5 | 59.1 |
|  | Microalbumin/Creatinine Ratio [mg/g; creat] | <3.7 | 18.8 | <10.8 | <4.2 | <13.6 | <3.5 | <10.4 | <9.0 | below quant | <5.1 |
|  |  |  |  |  |  |  |  |  |  |  |  |
| **Participant 7** | Specific Gravity | 1.015 | 1.021 | 1.021 | 1.025 | 1.018 | 1.019 | 1.023 | 1.018 | 1.021 | 1.018 |
|  | Creatinine [mg/dL] | 130 | 187 | 216.5 | 228.2 | 104.8 | 122.7 | 189.9 | 159.8 | 258.2 | 138.2 |
|  | Microalbumin/Creatinine Ratio [mg/g; creat] | 3.7 | 11.9 | 2.8 | 2.3 | 6.7 | 3.5 | 3.6 | 4.9 | 6.7 | 5.1 |
|  |  |  |  |  |  |  |  |  |  |  |  |
| **Participant 8** | Specific Gravity | 1.017 | 1.009 | 1.017 | 1.019 | 1.022 | 1.028 | 1.020 | 1.025 | 1.022 | 1.025 |
|  | Creatinine [mg/dL] | 120.1 | 28.7 | 106.3 | 129.1 | 88.2 | 217.6 | 115.7 | 271.9 | 118.4 | 237.2 |
|  | Microalbumin/Creatinine Ratio [mg/g; creat] | 3.9 | <10.5 | <2.8 | 2.7 | <3.4 | 2.6 | <2.6 | 7.6 | <2.5 | 3.0 |
|  |  |  |  |  |  |  |  |  |  |  |  |
| **Participant 9** | Specific Gravity | 1.016 | 1.018 | 1.025 | 1.024 | 1.020 | 1.022 | 1.019 | 1.020 | 1.016 | 1.013 |
|  | Creatinine [mg/dL] | 70.2 | 137.1 | 134.9 | 207.8 | 121.9 | 125.8 | 144.1 | 194.2 | 103.2 | 66.2 |
|  | Microalbumin/Creatinine Ratio [mg/g; creat] | <4.3 | <2.2 | 2.4 | 2.2 | 2.6 | <2.4 | 2.6 | 1.9 | <2.9 | <4.5 |
|  |  |  |  |  |  |  |  |  |  |  |  |
| **Participant 10** | Specific Gravity | 1.010 | 1.011 | 1.008 | 1.007 | 1.011 | 1.012 | 1.007 | 1.006 | 1.009 | 1.008 |
|  | Creatinine [mg/dL] | 24.2 | 28.2 | 21.1 | 32.7 | 24.2 | 47.0 | 16.6 | 14.7 | 23.2 | 15.9 |
|  | Microalbumin/Creatinine Ratio [mg/g; creat] | <12.4 | <10.6 | 28.0 | <9.2 | <12.4 | <6.4 | <18.1 | <20.4 | <12.9 | <18.9 |
